# Numerical investigation of the mixed-mode failure of snow

**DOI:** 10.1007/s40571-019-00224-5

**Published:** 2019-01-24

**Authors:** Dominika Mulak, Johan Gaume

**Affiliations:** 1 Imperial College London, London, UK; 2 SLAB Snow and Avalanche Simulation Laboratory, Swiss Federal Institute of Technology EPFL, Lausanne, Switzerland; 3 WSL Institute for Snow and Avalanche Research SLF, Davos, Switzerland

**Keywords:** Snow, Avalanche, Weak layer, Slab, DEM, Discrete element method, Cohesion, Sintering, Mixed-mode failure, Failure envelope

## Abstract

The failure of a weak snow layer underlying a cohesive slab is the primary step in the release process of a dry snow slab avalanche. The complex and heterogeneous microstructure of snow limits our understanding of failure initiation inside the weak layer, especially under mixed-mode shear–compression loading. Further complication arises from the dependence of snow strength on the loading rate induced by the balance between bond breaking and bond formation (sintering) during the failure process. Here, we use the discrete element method to investigate the influence of mixed-mode loading and fast sintering on the failure of a weak layer generated using cohesive ballistic deposition. Both fast and slow loading simulations resulted in a mixed-mode failure envelope in good agreement with laboratory experiments. We show that the number of broken bonds at failure and the weak layer strength significantly decreases with increasing loading angle, regardless of the loading rate. While the influence of loading rate appears negligible in shear-dominant loading (for loading angles above $30^{\circ }$), simulations suggest a significant increase in the weak layer strength at low loading angles and low loading rates, characteristic of natural avalanches, due to the presence of an active sintering mechanism.

## Introduction

**Fig. 1:**
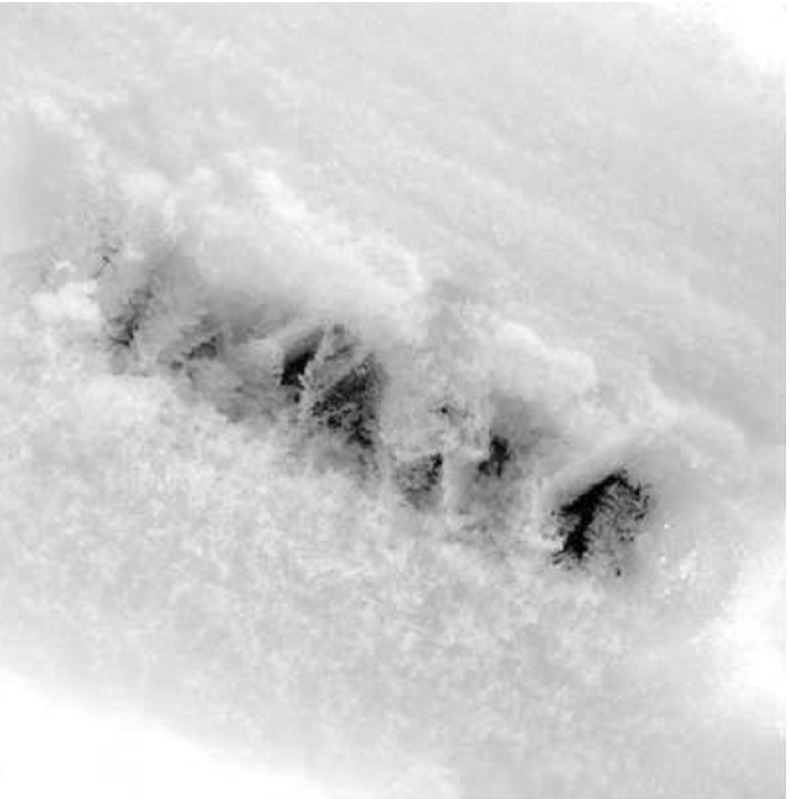
Buried surface hoar weak snow layer [16]. ©Jürg Schweizer

The origin of avalanche release can be traced back to a microscale crack which develops in a weak snow layer (Fig. 1) of significantly lower mechanical strength in comparison with the strongly bonded snow slab it is buried beneath [30, 32, 33]. Knowledge of the failure criterion of snow is crucial to avalanche hazard assessment, quantifying the maximum magnitude of stress a weak layer can withstand depending on the slab load and the slope angle. However, as the initial failure occurs on the individual bond scale, and owing to its microscale structural complexity, our understanding of the failure initiation process remains incomplete, especially under simultaneous shear and compressive loading and at different loading rates.

Several previous studies suggested a pure shear [19] or Mohr–Coulomb (MC) criterion for weak snow layer failure [6, 8–10, 14, 23, 24]. Under this model, failure is predicted to occur only for slope angles larger than the angle of internal friction, typically above $20^{\circ }$. More recently, the possible compressive failure of snow reported in laboratory experiments [5, 25, 35] was accounted for by including a compressive cap in the classical MC model leading to the so-called Mohr–Coulomb-Cap (MCC) model [25]. Despite a better understanding of snow mixed-mode failure, its micromechanical features (localized or diffuse failure [22]) are still not fully understood.

**Fig. 2:**
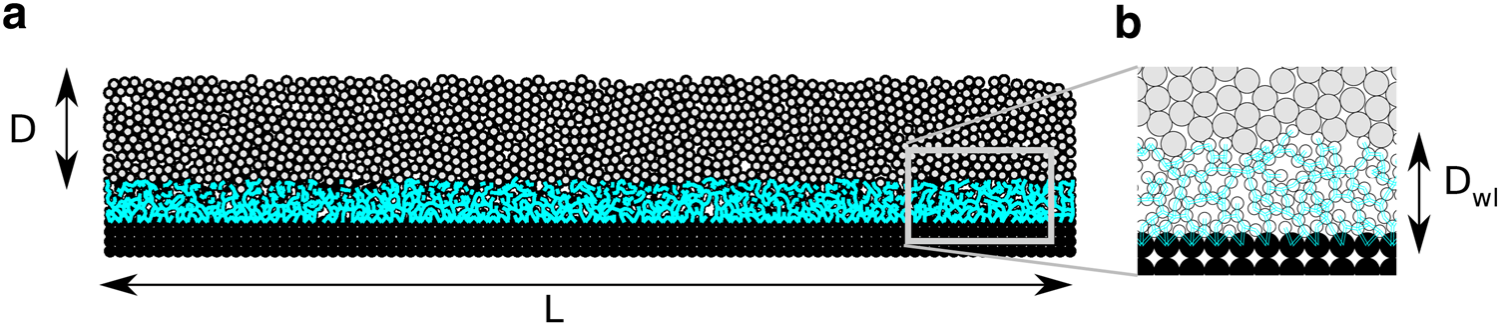
**a** The simulated weak snow layer (blue)—slab (gray) system. **b** Magnified image of the weak layer; cohesive bonds between numerical grains are shown in blue. The left and right sides are free (no confinement), and the basal layer (black) is fixed. (Color figure online)

A further hindrance to a set of clear snow failure conditions arises from its rapidly changing nature and therefore its dependence on the rate at which it is loaded. Snow is classified as a “hot” material, existing naturally at a temperature close to its melting point. Under sufficient compressive force, broken or new contacts between ice grains can rebond in the fast sintering process [36]. Laboratory experiments at different loading rates have shown the importance of sintering on the snow strength [23, 25–28, 31]. In the field, the recent experiments of Birkeland et al. [2] and Schweizer et al. [34] showed the complex interplay between time and loading for the evaluation of snowpack stability. In particular, whereas increasing load over short time frames (< 1 h) induced a decrease in stability, the latter significantly increased over longer time frames (15 mn to 4 days) [2]. Sintering in snow is thought to explain the commonly observed transition of snow mechanical behavior from ductile at low strain rates to brittle at very high strain rates. Experiments of Schweizer [31] demonstrate a clear brittle failure behavior when the snow sample is strained at a high rate, while at very low strain rates the internal stress in the material continues to increase even for large deformations. This delays the global failure of the material and increases its strength. This ductile-to-brittle transition coupled with a strain-softening model allowed Barraclough et al. [1] to reproduce the propagation and reflection of compaction bands in the confined compression of snow.

Artificial or accidental avalanches are most often triggered by the near-instantaneous application of force, for example by a skier, at rates larger than around 0.1–1 kPa/s [29]. At these fast loading rates, based on the previously mentioned experiments, it is assumed that the onset of catastrophic failure may occur too quickly for the sintering of broken bonds to affect the failure behavior of the weak layer.

In contrast, natural avalanches occur due to the gradual accumulation of snow mass above the weak layer, thus at a much slower loading rate (typically less than $\approx 1$Pa/s [29]). In such conditions, sintering effects become important. The more gradual damage at a slow loading rate allows the sintering mechanism to significantly strengthen and heal bonds during loading.

Recent work has evidenced the promise of the discrete element method (DEM) in leading to a more complete understanding of the micromechanics of porous cohesive materials and more generally avalanche release [3, 12, 15–17]. Moreover, DEM has shown promise as a suitable means for the development of homogenized constitutive laws for large-scale models [11, 18, 20, 21]. Here, in order to better understand slab avalanche release in varying conditions of slope angle and loading rate, we developed a discrete element snow model with a simplified porous weak layer generated using cohesive ballistic deposition. Contacts between particles are represented in the model by cohesive bonds which are allowed to break or heal under different loading rates at different characteristic times. We investigate the failure conditions of weak snow layers under mixed-mode loading through load-controlled simulations corresponding to fast and slow loading rates.

## Methods: a cohesive discrete element model

### Simulation setup

We use the discrete element method (DEM) for simulating the mechanical response of the weak snow layer under an applied load, at or close to the point of failure. DEM simulations were performed using the commercial software PFC2D (by Itasca) in which the original soft-contact algorithm described in [7] is available. The simulated two-dimensional (plain strain) system (Fig. 2) is composed of a completely rigid basal layer, a weak layer (WL) of thickness $D_{\mathrm {wl}}$ and an overlying rigid slab of thickness $D = 0.2\,\hbox {m}$. The slab, whose sole function is to apply a uniform load onto the weak layer below, is composed of grains of radius $r = 0.01\,\hbox {m}$ and was generated using random pluviation (without cohesion) also known as ballistic deposition. The weak layer is composed of grains of radius $r_{\mathrm {wl}} = \frac{r}{2}$ and is generated by random cohesive pluviation; randomly distributed particles are submitted to gravity and any new contacts formed during the particles’ free fall result in cohesive bonds. The numerical grains are not intended to represent real snow grains, which are generally smaller in the slab than in the weak layer, but serve as numerical tools necessary for the analysis of realistic interactions and properties in the weak layer. Hence, the choice to have larger particles in the slab than in the weak layer (which was also done in [15, 16]) is made to improve the computational time of the simulations, given that the role of the slab is only to apply the load on the weak layer.

### Contact model

We use the same interparticle contact model as described in Gaume et al. [12, 15, 16], the so-called parallel-bond model. This model consists of two parts acting in parallel:

- a standard linear contact model with a constant elastic modulus *E*, Poisson’s ratio $\nu$ and friction coefficient $\mu$;
- a cohesive bond which can be envisioned as a point of glue with constant elastic modulus $E_\mathrm {b}$ and Poisson’s ratio $\nu _\mathrm {b}$ acting at the contact points. This bond has a specified tensile and shear strength, $\sigma ^{\mathrm {max}}_\mathrm {b}$ and $\tau ^{\mathrm {max}}_\mathrm {b}$. The bond can break under shear, tension and bending according to beam theory.

More details about this parallel-bond model can be found in Gaume et al. [12, 15, 16]. Note that we prescribed the strength of the interface between the weak layer and the slab at an infinite value to enforce the failure to occur within the weak layer (Table 1).

**Table 1:** Contact model parameters

Parameter	WL	Interfaces
*E*	$10 \times 10^{6}\hbox { Pa}$	$10 \times 10^{6}\hbox { Pa}$
$\nu$	0.3	0.3
$\mu$	0.5	0.5
$E_\mathrm {b}$	$10 \times 10^{6}\hbox { Pa}$	$10 \times 10^{6}\hbox { Pa}$
$\nu _\mathrm {b}$	0.3	0.3
$\sigma ^\mathrm {max}_\mathrm {b}$	$1 \times 10^{6}\hbox { Pa}$	$\infty$
$\sigma ^\mathrm {max}_\mathrm {b}/\tau ^\mathrm {max}_\mathrm {b}$	2	2

*WL* weak layer, *Interfaces* interfaces between the slab and the weak layer and between the weak layer and the base

### Mixed-mode loading and stress measures

A uniform load is applied onto the weak layer by progressively increasing the density of the overlying rigid slab at each time step during the simulation, until onset of catastrophic failure occurs. Mixed-mode simulations were carried out for different loading angles $\psi$ corresponding to the orientation of gravity according to $g_x=g\cos \psi$ and $g_z=-g\sin \psi$ (*x*: horizontal direction; *z*: vertical direction). Simulations are performed for loading angles $\psi$ between 0 and 180$^\circ$ leading to mixed-mode shear–compression or shear–tension loading states.

We define the total applied stress $\sigma _{\mathrm {app}}(t)=\rho (t)gD$ (increasing slab load). The loading rate was chosen to avoid inertial effects before failure ${\dot{\sigma }_\mathrm {{app}}}(t)=0.004/\Delta t$ (Pa/s) ($\Delta t$ is the time step of the simulation).

The shear stress $\tau$ and normal stress $\sigma$ in the weak layer are measured based on the sum of the shear and normal forces at the interface between the weak layer and the base as follows (in 2D):

1 $$\begin{aligned} \tau =\frac{1}{L}\sum _{i\in \mathcal {I}}F^i_\mathrm {s}, \end{aligned}$$

and

2 $$\begin{aligned} \sigma =\frac{1}{L}\sum _{i\in \mathcal {I}}F^i_\mathrm {n}, \end{aligned}$$

where $F^i_\mathrm{s}$ and $F^i_\mathrm{n}$ are the shear and normal forces for contact *i* and $\mathcal {I}$ is the subset of contacts between the weak layer and the base. Based on the shear and normal stresses at the bottom of the weak layer, we define the total weak layer stress as

3 $$\begin{aligned} \sigma _{\mathrm {tot}}=\sqrt{\tau ^2+\sigma ^2}. \end{aligned}$$

Similarly, we define the average weak layer shear strain $\gamma$ and normal strain $\epsilon$ based on the average slab displacement ${\varvec{u}}$ as

4 $$\begin{aligned} \gamma =\frac{u_x}{D_\mathrm {wl}}, \end{aligned}$$

and

5 $$\begin{aligned} \epsilon =\frac{u_z}{D_\mathrm {wl}}, \end{aligned}$$

and the total weak layer strain as

6 $$\begin{aligned} \epsilon _{\mathrm {tot}}=\sqrt{\gamma ^2+\epsilon ^2}. \end{aligned}$$

Finally, we also define the average slab velocity $v_\mathrm {s}$ as

7 $$\begin{aligned} v_\mathrm {s}=||{\varvec{\dot{u}}}||. \end{aligned}$$

Failure is identified using a two-step criterion. First, a criterion based on the average velocity of the slab, which strongly increases after failure, allows us define a lower range of deformation for the search. Second, we identify the maximum value of $\sigma _\mathrm {tot}$ within this range. In our simulations (see below), we found that a critical velocity threshold of 0.2 m/s leads to accurate failure detection. Finally, the total strength is defined as the total stress in the weak layer $\sigma _{\mathrm {tot}}$ at failure; the compressive and tensile strengths correspond to the normal stress $\sigma$ at failure for loading angles of $0^\circ$ (pure compression) and $180^\circ$ (pure tension), respectively; the shear strength corresponds to the shear stress $\tau$ at failure for a loading angle of $90^\circ$ (pure shear).

**Fig. 3:**
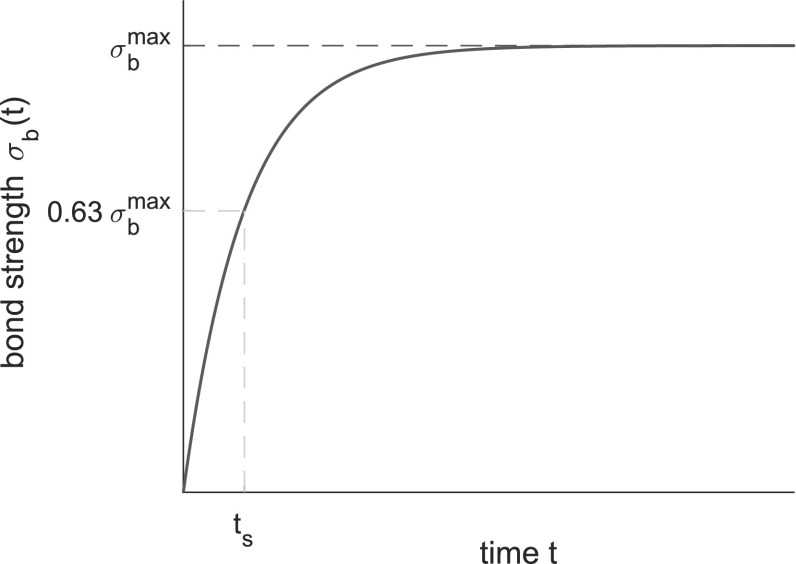
The sintering model (Eq. 8)

**Fig. 4:**
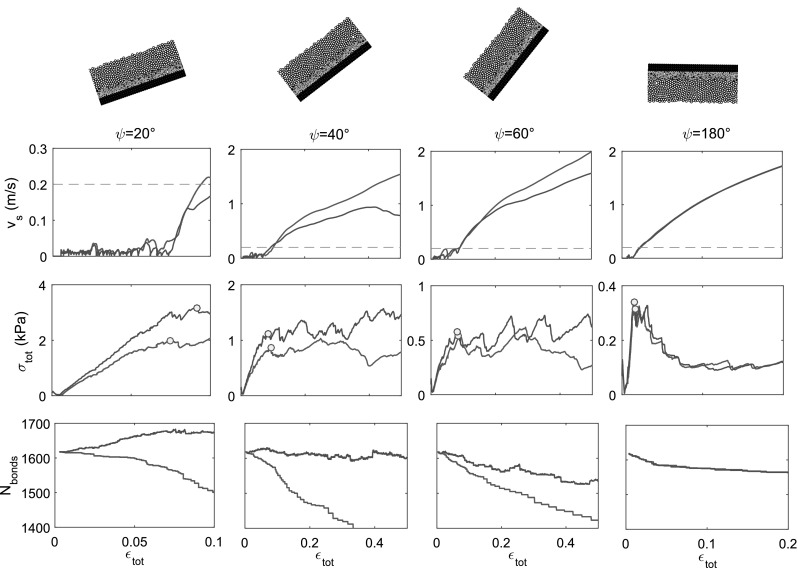
(Top) slab velocity as a function of total strain. (Center) Weak layer total stress as a function of total strain. (Bottom) Number of cohesive bonds as a function of total strain. The different columns (left to right) correspond to different loading angles illustrated above the plots. Blue: without sintering. Red: with sintering and $t_\mathrm {s}=300\Delta t$. (Color figure online)

### Implementation of sintering

We implement the sintering mechanism in weak layer particle interactions through the installation of a bond at new points of contact, whose strength evolves in time according to an exponential model, as shown in Fig. 3, similar to the sintering fiber bundle model developed by Reiweger et al. [28]. The key model parameters characterizing the sintering process are the sintering time $t_\mathrm{s}$ taken for the strength $\sigma _\mathrm {b}$ of the new bond to reach $0.63 \sigma ^\mathrm {max}_\mathrm{b}$. The sintering mechanism was activated only for positive values of the normal force $F_\mathrm{n}$ between unbonded contacts. Shear and normal bond strength increases in the new contacts as a function of contact time according to equations 8 and 9, where $\sigma _\mathrm {b}$ and $\tau _\mathrm {b}$ are the normal and shear components of the bond strength, respectively.

8 $$\begin{aligned} \sigma _\mathrm {b}(t) = \sigma _\mathrm {b}^\mathrm {max}\left( 1 - e^{-\frac{t}{t_\mathrm {s}}}\right) , \end{aligned}$$

and

9 $$\begin{aligned} \tau _\mathrm {b}(t) = \tau _\mathrm {b}^\mathrm {max}\left( 1 - e^{-\frac{t}{t_\mathrm {s}}}\right) . \end{aligned}$$

In nature, the sintering time is an intrinsic material property, on the order of $t_\mathrm {s} \sim 1$ s for snow [36] and is itself independent of the loading rate. However, we want to avoid potential numerical instabilities due to inertial effects as a result of a changing loading speed during simulations. Hence, the system was always loaded at a constant rate for which the results without sintering are not found to depend on the loading rate. The influence of sintering was thus introduced by modifying the sintering time $t_\mathrm {s}$ in order to represent the fast and slow loading scenarios. Hence, $t_\mathrm {s}\rightarrow \infty$ represents very fast loading (no sintering), while $t_\mathrm {s}\rightarrow 0$ represents very slow loading (immediate strength recovery at contact).

### Experimental data

For model validation, we use the laboratory experiments performed and described in details by Reiweger et al. [25]. Detailed information about the loading apparatus can be found in [29]. We recall here the main characteristics of the data. Weak layers of surface hoar (natural), depth hoar and faceted crystals (natural and artificial) were loaded at different loading angles ranging from $0^\circ$ to $35^\circ$. Experiments were performed at loading rates between 1 Pa/s (intense snowfall or wind loading) and 440 Pa/s (artificial loading).

**Fig. 5:**
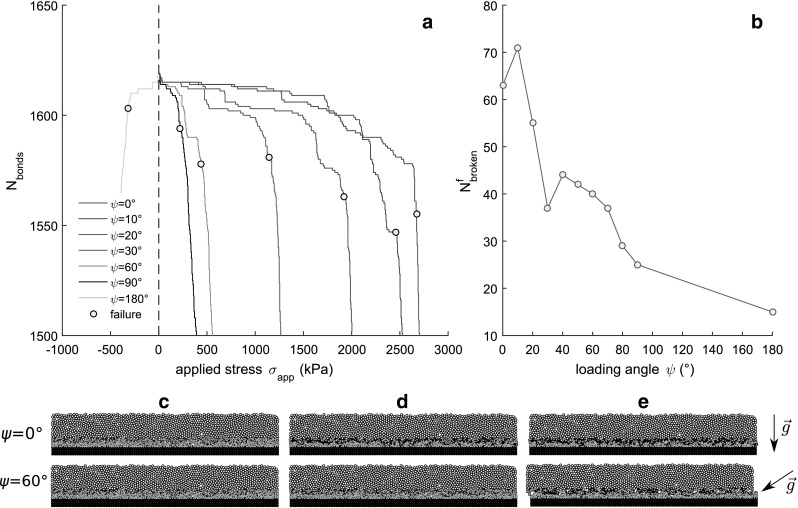
**a** Number of cohesive bonds as a function of the applied stress. **b** Number of broken bonds at failure as a function of loading angle. **c** Initial sample state **d**) at failure **e** postfailure. Results of simulations without sintering

## Results

### Simulations without sintering

Simulations had the same general characteristics, regardless of the loading angle (Fig. 4). Before failure, we observe a relatively small decrease in the number of cohesive bonds $N_\mathrm {bonds}$ associated with an almost linear increase in the total stress with increasing total strain and a very small slab velocity. However, we also observe that local bond breaking events can lead to small stress variations as well as velocity bursts, even before catastrophic failure. This typical behavior is nicely observed in Fig. 4 for a loading angle $\psi =20^\circ$. After failure, we observe an important decrease in the number of cohesive bonds (see also Fig. 5a) which is generally associated with a decrease in the total stress, i.e., a strain-softening behavior. The amount of softening increases with increasing loading angle, with a maximum drop in total stress observed for a pure tension simulation, i.e., $\psi =180^\circ$. For low values of the loading angle, there is almost no softening and the total stress levels off after failure. In addition, Fig. 5 shows that the number of broken cohesive bonds required for catastrophic failure decreases significantly with increasing loading angle, with a minimum found for $\psi =180^\circ$. In more detail (Fig. 5b), the number of broken bonds required for catastrophic failure in pure compression ($\psi =0^\circ$) is approximately five times larger than that for pure tension ($\psi =180^\circ$) and three times larger than for pure shear ($\psi =90^\circ$).

**Fig. 6:**
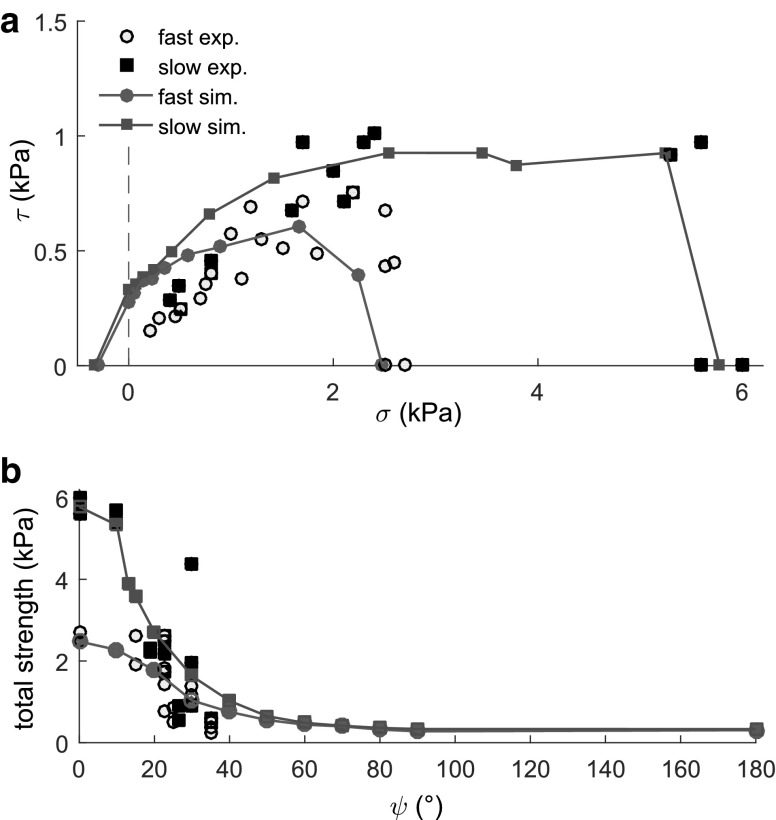
**a** Failure envelope corresponding to “fast” simulations without sintering (blue) and “slow” simulations with sintering (red). Data of Reiweger et al. [25] for fast (circles) and slow (squares) loading. **b** Total strength of the samples versus loading angle. Colors and symbols identical as a. (Color figure online)

We obtain the failure envelope of the weak layer [25] by analyzing the dependency of the shear stress $\tau$ on the normal stress $\sigma$ (Fig. 6a). For low values of the normal stress ($<1.8$ kPa), corresponding to large loading angles ($>20^{\circ }$), we observe an increase in the shear stress with increasing normal stress. However, for larger normal stresses and thus lower loading angles, the shear stress decreases with increasing normal stress to allow for failure under compression. This behavior leads to a slow decrease in the weak layer total strength with increasing loading angles for angles $<20^{\circ }$, followed by a sharp decrease for larger values.

As shown in Fig. 6a the shape of our simulated failure envelope is in good agreement with that based on laboratory experiments performed at high loading rates [25]. Quantitatively, we slightly overestimate the shear stress at failure for loading angles between 30 and $40^{\circ }$. Note that the bond strength $\sigma ^\mathrm {max}_\mathrm {b}$ was chosen to match the compressive strength obtained in the fast experiments of [25].

### Simulations with sintering

Simulations with sintering were performed under the same conditions and contact properties as without sintering. The main difference is that new contacts can heal, with a bond normal and shear strength which evolves in time according to Eqs. 8 and 9. The sintering time $t_\mathrm {s}$ was chosen so as to reproduce the compressive strength obtained in the slow experiments of [25]. We found that a sintering time of $t_\mathrm {s}=300\Delta t$ led to good agreement with laboratory data.

The general characteristics of the sintering simulations are generally very similar to those without sintering (Fig. 4). The main difference concerns the critical number of cohesive bonds and the stress in the weak layer which are larger, especially for low values of the loading angle. For a purely tensile loading case (Fig. 4, $\psi =180^\circ$), the formation of new contacts is very unlikely so simulations with sintering yield the same results as without sintering. However, a decrease in the loading angle leads to a larger total stress and number of cohesive bonds before, at and after failure. In some cases (Fig. 4, $\psi \le 40^\circ$), the number of cohesive bonds becomes larger than the initial value.

The simulated failure envelope with sintering has a very similar shape to that without sintering (Fig. 6a). The effect of sintering is significant only for loading angles $\le 40^\circ$. The compressive strength is increased by a factor of 2.2 compared to the case without sintering. Sintering induces a stronger decrease in the total strength with increasing loading angle (Fig. 6b) which tends to the value without sintering for a pure tension loading case. Hence, the role of sintering appears highly significant in compression-dominated loading modes and leads to large differences in the strength of the weak layer for loading angles typically lower than $40^\circ$. Similar to the case without sintering, the simulated failure envelope is in very good agreement with the laboratory experiments of Reiweger et al. [25] performed at low loading rates, allowing an active sintering mechanism. Similar to the case without sintering, the strength for loading angles between 30 and $40^{\circ }$ is slightly overestimated by the model.

Finally, we performed simulations with different values of the sintering time $t_\mathrm {s}$. These simulations highlighted that, for $t_\mathrm {s}>30000\Delta t$, the results with sintering yielded the exact same results as without sintering, meaning that catastrophic failure occurred faster than the time required to form new cohesive contacts.

### Rate dependence

Our simulations were performed at a constant rate $\dot{\sigma }_\mathrm {{app}}(t)=0.004/\Delta t$ for different sintering times $t_\mathrm {s}$. This choice was made to prevent the influence of inertial effects on the strength of our samples, i.e., so that the strength of samples without sintering is uninfluenced by the loading rate. However, the characteristic time for fast sintering of snow was shown to be close to 1 s [36]. Hence, it is possible to interpret our results in a different manner, i.e., with a constant sintering time $t_\mathrm {s}$ and different loading rates, to compare to real-world values. In the case with sintering for $t_\mathrm {s}=300\Delta t$, the applied stress increment during sintering is equal to 1.2 Pa. Hence, for a sintering time of 1 s, this would correspond to a loading rate of 1.2 Pa/s, corresponding to an intense natural snowfall. As a consequence, we refer to this case as a “slow” simulation (similar to [25, 26]). On the other hand, we found that simulations performed for a sintering time $t_\mathrm {s}>30000\Delta t$ were exactly the same as those without sintering. The applied stress increment during this sintering time is equal to 120 Pa. Hence, for a sintering time of 1 s, we find that the effects of sintering become negligible for loading rates higher than 120 Pa/s, characteristic of the rates induced by a skier or a snowmobile. We refer to these cases as “fast” simulations (similar to [25, 26]).

## Discussion

DEM loading simulations of a weak layer formed by cohesive ballistic deposition and buried under a cohesive slab layer resulted in a modified mixed-mode failure envelope with failure modes in tension, shear and compression. Simulated failure envelopes obtained with or without sintering were in good agreement with data from laboratory experiments of snow failure performed for fast or slow loading, respectively, and also with the Mohr–Coulomb-Cap (MCC) model proposed by Reiweger et al. [25]. Regardless of the effect of sintering, the total strength of the weak layer strongly decreases with increasing loading angle. In addition, the number of broken cohesive contacts required for catastrophic failure strongly decreases with increasing loading angle. In fact, the difference in strength between different loading angles appears to be directly related to the number of broken bonds (Figs. 5a, 6b). More specifically, for fast simulations the ratio of the compressive to tensile strength is approximately equal to the ratio between broken bonds in compression and in tension ($\sim 5$).

The introduction of sintering, through the formation of new bonds of increasing strength with time, led to a significant increase in the shear and normal stress for loading angles typically below 40$^\circ$. For very large loading angles, the effect of sintering was negligible. In addition, by assuming a sintering time of 1 s and a loading rate of $\sim$1 Pa/s, we were able to reproduce the “slow” experiments of Reiweger et al. [25]. In contrast, “fast” experiments were reproduced for loading rates typically larger than 100 Pa/s. Hence, our model successfully captures the so-called strain-rate dependency of snow [31]. This increase in snow strength with decreasing loading rate was also well captured by the fiber bundle model of Reiweger et al. [28] and Capelli et al. [4] (also including viscous stress relaxation), but they did not investigate the effect of the loading angle and sintering, simultaneously.

In view of snow slab avalanche release, we showed that the shear strength on typical avalanche slopes ($30\le \psi \le 45^\circ$, [32]) was significantly lower ($\sim$ 5 times) than the compressive strength, for both slow and fast simulations. Hence, although the failure is obviously induced under a mixed-mode loading state, the shear component of the stress has a much larger influence on failure. Concerning the rate dependency, although fast sintering has apparently no effect on artificial loading such as by a skier [13] or even a Propagation Saw Test [15, 16, 37], it would certainly influence natural avalanche release as the weak layer would gain strength with time and loading during a snowfall or a strong wind episode. For instance, if a weak layer was loaded infinitely fast due to a snowfall of density $\rho =100$ kg/m$^3$ on a 35$^\circ$ slope, it would fail for a thickness of the snowpack above the weak layer of $\sim$ 1 m (calculated using the “fast” failure envelope). On the other hand, if the snowpack is loaded at a rate of $\sim$ 1 Pa/s, the same weak layer would fail for a slab thickness of $\sim$ 1.6 m (calculated using the “slow” failure envelope).

Recent work of Gaume et al. [15, 16] suggested that a very simplified triangular weak layer structure was sufficient to capture the main ingredient required for dynamic crack propagation (mixed-mode failure and collapse). However, the shape of the failure envelope of this weak layer structure was not in good agreement with laboratory data. In addition, recent DEM simulations by Hagenmuller et al. [17] and Gaume et al. [12] suggested that the main drivers of snow microstructure failure were the volume fraction and the cohesive coordination number (number of cohesive contacts per particle). Although such microstructural descriptions would surely improve our results, the latter studies suggest that a simplified structure could be relevant. As a consequence, we decided to improve the oversimplified structure of Gaume et al. [15] by creating a weak layer by cohesive ballistic deposition. Our results suggest that this simplified structure is sufficient to reproduce the mixed-mode failure behavior of weak snow layer under different loading rates.

One important limitation of our study is the two-dimensional character which prevents reaching a large porosity for our modeled weak layer based on cohesive ballistic deposition. Here, the porosity of the weak layer is around 0.4, which is almost almost half of the value of the weak layers modeled in [12, 15–17, 38]. Although this limitation does not influence failure initiation and the mixed-mode failure envelope, which were well reproduced, it strongly influences the postpeak behavior, i.e., strain-softening and volumetric collapse of the weak layer which would be strongly underestimated in our case (see, e.g., Fig. 4). A more porous structure would facilitate collapse of the weak layer during loading as particles would be able to accommodate gaps created as a result of shearing of the weak layer. However, our 2D simulations including sintering for low values of the loading angle required significant computational resources which is why we focused on the two-dimensional case to harvest the influence of sintering and loading angle on failure initiation. In the future, three-dimensional simulations using the same technique to create the weak layer will lead to more realistic porosity values and will allow us to study not only failure initiation, but also dynamic crack propagation [3]. It could also allow us to study the micromechanics associated with the propagation and reflection of compaction bands in snow [1].

## Conclusions

The discrete element method was used to investigate the failure behavior of a simplified weak snow layer model and its dependence on loading rate. The failure envelope, derived from a series of mixed-mode loading simulations, was found to be in good agreement with experimental results on snow failure performed at different loading rates and with the modified Mohr–Coulomb-Cap failure criterion proposed by Reiweger et al. [25]. We showed that the number of bonds required for catastrophic failure decreases significantly with the loading angle. In particular, the number of broken bonds at failure was largest for simulations under compression, where the failure appeared to be diffuse, while it was significantly lower for cases under shear and tension where the failure appeared to be localized.

The effect of loading rate on the failure behavior appears negligible for shear-dominated loading modes (slope angles $\psi \ge 30^{\circ }$) in comparison with compression-dominated loading, where the weak layer may gain in strength when loaded slowly due to the dominance of the sintering mechanism over bond breaking at slow loading rates, typically of the order of 1 Pa/s. Results obtained for simulations performed at loading rates larger than 100 Pa/s were the same as those obtained without sintering.

The proposed approach should be extended in the future to three dimensions in order to reach a more realistic porosity of the weak layer in view of modeling crack propagation which is strongly influenced by the collapse of the weak layer [3].
